# The Relationship between National-Level Carbon Dioxide Emissions and Population Size: An Assessment of Regional and Temporal Variation, 1960–2005

**DOI:** 10.1371/journal.pone.0057107

**Published:** 2013-02-20

**Authors:** Andrew K. Jorgenson, Brett Clark

**Affiliations:** 1 Department of Sociology, University of Utah, Salt Lake City, Utah, United States of America; 2 Environmental & Sustainability Studies Program, University of Utah, Salt Lake City, Utah, United States of America; The Ohio State University, United States of America

## Abstract

This study examines the regional and temporal differences in the statistical relationship between national-level carbon dioxide emissions and national-level population size. The authors analyze panel data from 1960 to 2005 for a diverse sample of nations, and employ descriptive statistics and rigorous panel regression modeling techniques. Initial descriptive analyses indicate that all regions experienced overall increases in carbon emissions and population size during the 45-year period of investigation, but with notable differences. For carbon emissions, the sample of countries in Asia experienced the largest percent increase, followed by countries in Latin America, Africa, and lastly the sample of relatively affluent countries in Europe, North America, and Oceania combined. For population size, the sample of countries in Africa experienced the largest percent increase, followed countries in Latin America, Asia, and the combined sample of countries in Europe, North America, and Oceania. Findings for two-way fixed effects panel regression elasticity models of national-level carbon emissions indicate that the estimated elasticity coefficient for population size is much smaller for nations in Africa than for nations in other regions of the world. Regarding potential temporal changes, from 1960 to 2005 the estimated elasticity coefficient for population size decreased by 25% for the sample of Africa countries, 14% for the sample of Asia countries, 6.5% for the sample of Latin America countries, but remained the same in size for the sample of countries in Europe, North America, and Oceania. Overall, while population size continues to be the primary driver of total national-level anthropogenic carbon dioxide emissions, the findings for this study highlight the need for future research and policies to recognize that the actual impacts of population size on national-level carbon emissions differ across both time and region.

## Introduction

Population size and growth are widely recognized as primary drivers of national-level anthropogenic carbon dioxide emissions, other greenhouse gas emissions, and environmental degradation outcomes, including ground-level air pollutants, deforestation, and the overall consumption of natural resources [1], [2], [3], [4], [5]. Indeed, the scientific and policy communities as well as governmental and intergovernmental agencies throughout the world place great emphasis on the role of population when considering the human dimensions of global climate change and other global and regional ecological disruptions [6], [7], [8]. In April 2012, the Royal Society published a report, *People and the Planet*, indicating that population growth and rapidly increasing levels of consumption are exhausting the world's resources and producing an array of environmental ills, such as lack of fresh water and the accumulation of carbon dioxide in the atmosphere [9].

While such a general emphasis on environment and population relationships seems logical, with relatively few exceptions [10], [11] it is implicitly assumed that the actual impact of population size on carbon emissions and other similar outcomes is uniform in magnitude across time and location. For example, the STIRPAT orientation (“Stochastic Impacts by Regression on Population, Affluence and Technology”) is perhaps the most well established and widely known body of work in the environmental social sciences that routinely considers in cross-national analyses the effects of population size on greenhouse gas emissions as well as other related sustainability factors and environmental change conditions (see http://stirpat.msu.edu/ for the extensive bibliography of published research in this multidisciplinary tradition). This body of research consistently shows that the effect of population size on national-level total carbon emissions is positive and much larger in magnitude than other human drivers, such as economic development and levels of urbanization (e.g., [12], [13]). However, the published cross-national longitudinal research in the STIRPAT tradition on human drivers of national-level greenhouse gas emissions models the effect of population size as being time invariant, and both the longitudinal and cross-sectional studies rarely consider regional-level variations in relationships between national-level environmental outcomes and population size (e.g., [2]). Environmental economists have also conducted cross-national longitudinal analyses of the effects of population size on the carbon emissions of nations, but like the STIRPAT research tradition, they tend to model the effect of population size as time-invariant and fail to adequately consider regional-level differences [14]. Researchers in the health sciences have conducted descriptive analyses of the global carbon emissions and global population relationship, but such studies focus on the similarities in plotted growth for both, and neglect to include critical control variables, such as economic development and urbanization [15]. Further, such global-level analyses are unable to identify regional-level differences, and they do not provide estimated coefficients for the effects of population parameters or other factors on carbon emissions.

Some econometrics modeling techniques, such as general equilibrium models, are able to model relationships between population and carbon emissions through a complex socio-economic system which is characterized by time-variant and regional-specific market structures, household characteristics, and energy technologies. The actual effects of population size on national-level carbon emissions and whether or not they change through time are not the focus of the research that uses this modeling approach. Rather, research on carbon emissions in this tradition commonly focuses on the effects of a wide range of policies for international trade and financial flows, while taking into account the role of population size and other socioeconomic factors [16], [17]. While such model estimation techniques are rigorous and useful for various areas of econometrics inquiry, they do not provide relatively direct and easily interpretable estimated coefficients for the effects of independent variables on the modeled outcome.

To determine if the related assumptions about the similarity and temporal stability of the national-level carbon emissions/population size relationship are in fact a reality requires a rigorous comparative-international and longitudinal analysis of this relationship that employs appropriate measures and panel regression modeling techniques that allow for the assessment of potential regional and temporal variations. As important, employed statistical techniques for this sort of inquiry need to balance methodological rigor with relatively clear and straightforward interpretations [18]. Such rigorous and interpretable modeling techniques have been successfully employed in recent cross-national longitudinal research on the temporal and regional variations in the effects of economic development on total carbon emissions, per capita carbon emissions, and emissions per unit of production [19], [20], [21] as well as in cross-national research on the effects of development and sector-specific production activities on anthropogenic methane emissions [11].

Through time, changes in technology, built infrastructure, social institutions, ecological conditions, culture, and individual behaviors could modify the magnitude of the influence of population parameters on environmental outcomes, including carbon emissions. And such technological, ecological, and social conditions often vary by place and time, thereby potentially leading to observed regional-level and/or temporal differences in relationships between national-level total carbon emissions and population size [4]. Given the importance in understanding the human drivers of national-level greenhouse gas emissions, the attention placed on the role of population size, and the consistent finding in past research that population is a primary driver of national-level emissions, in this study we conducted cross-national longitudinal analyses of 85 countries from 1960 to 2005 to assess the common assumption that the effect of population size on national-level carbon dioxide emissions is similar in size across macro-regions and through time.

The study consisted of multiple steps, with the findings discussed in the results section below. First, as an important preliminary descriptive analysis, we assessed the general changes in total carbon dioxide emissions levels and population size from 1960 to 2005 for the overall sample of nations as well as for samples of nations by macro-region, including the regions of (1) Africa, (2) Asia, (3) Latin America, and (4) Europe, North America, and Oceania combined. Next, we employed well-established rigorous two-way fixed effects panel regression analysis techniques to estimate elasticity coefficients for the effect of national-level population size on national-level total carbon dioxide emissions, net of various controls established in the literature on human drivers of emissions, and we continued by assessing if the estimated effect of population size on national-level carbon dioxide emissions varies by region. In the final steps of the study, for the entire sample of nations as well as for samples restricted to each region we estimated panel regression elasticity models to assess if the effect of population size on national-level total carbon dioxide emissions changes in value through time. Table 1 lists the 85 countries included in the study.

**Table 1:** Countries included in the study.

		*Europe, North America,*
*Africa*	*Asia*	*and Oceania*
Algeria	Bangladesh	Australia
Benin	China	Austria
Burkina Faso	India	Belgium
Burundi	Indonesia	Canada
Cameroon	Iran	Denmark
Central African Republic	Israel	Finland
Chad	Japan	France
Congo, Dem. Rep.	Korea, Rep.	Georgia
Congo, Rep.	Malaysia	Greece
Cote d'Ivoire	Nepal	Hungary
Egypt	Pakistan	Ireland
Ghana	Philippines	Italy
Kenya	Sri Lanka	Latvia
Liberia	Syrian Arab Republic	Mexico
Madagascar	Thailand	Netherlands
Malawi		New Zealand
Mauritania	*Latin America*	Norway
Morocco	Argentina	Papua New Guinea
Niger	Bolivia	Portugal
Rwanda	Brazil	Spain
Senegal	Chile	Sweden
Sierra Leone	Colombia	Switzerland
South Africa	Costa Rica	United Kingdom
Sudan	Dominican Republic	United States
Togo	Ecuador	
Tunisia	El Salvador	
Zambia	Guatemala	
Zimbabwe	Haiti	
	Honduras	
	Nicaragua	
	Panama	
	Paraguay	
	Peru	
	Uruguay	
	Venezuela	

## Results

For the overall sample of 85 countries, their combined total carbon dioxide emissions increased from 6,172,206,000 metric tons in 1960 to 21,740,550,000 metric tons in 2005, which corresponds to slightly over a 252% increase. Their combined population size increased from 2,377,785,191 people in 1960 to 5,166,206,465 people in 2005, equivalent to slightly above a 117% increase. For the sample of 28 countries in Africa, their combined total emissions increased from 147,396,000 metric tons in 1960 to 748,816,000 metric tons in 2005, equivalent to slightly over a 408% increase. Their combined population size increased from 169,788,509 people in 1960 to 534,440,871 people in 2005, corresponding to slightly under a 215% increase. Turning to the sample of 15 countries in Asia, their combined carbon dioxide emissions increased from 1,264,603,000 metric tons in 1960 to 10,095,701,000 metric tons in 2005, while their combined population size increased from 1,522,309,168 to 3,406,914,103. The former represents approximately a 698% increase, while the latter represents slightly less than a 124% increase. For the sample of 18 countries in Latin America, their combined emissions increased almost 443% from a value of 169,784,000 metric tons in 1960 to 921,749,000 metric tons in 2005. Their combined population size increased from 167,262,577 people in 1960 to 430,180,395 people in 2005, an increase of slightly over 157%. Turning to the sample of 24 countries in Europe, North America, and Oceania, their combined carbon dioxide emissions increased from 4,590,423,000 metric tons in 1960 to 9,974,284,000 metric tons in 2005, representing slightly more than a 117% increase. Their combined population size was 518,424,937 people in 1960 and increased to 794,671,097 people in 2005, which marks approximately a 53% increase.

We now turn to the findings for the panel regression analyses. Table 2 reports the results of two-way fixed effects panel models of total carbon dioxide emissions for the overall sample of 85 nations from 1960 to 2005. As explained in the Materials and Methods section below, the two-way fixed effects are accounted for by the inclusion of dummy variables for each case (i.e., country) as well as dummy variables for each time point (i.e., year of observations). Model one includes population size as well as controls for gross domestic product (GDP) per capita and GDP per capita squared to account for a potential Kuznets curve, urban population as a percent of total population, and trade as a percent of total GDP. These additional predictors are well established in the existing literature on the human drivers of greenhouse gas emissions [1]. The second model also includes interactions between population size and region (Africa, Asia, Latin America), with the combined region of Europe, North America, and Oceania serving as the reference group. These interactions allow for assessing if the estimated effect of population size on carbon dioxide emissions varies by region. It is important to note that the main effects of the dummy variables for the category are excluded from the model since they are perfectly correlated with and thus accounted for by the case-specific fixed effects [11]. All explanatory variables and the dependent variable are in logarithmic (ln) form. This approach is known in statistics and econometrics as an elasticity model [22]. It is a statistical modeling technique that is commonly employed in research on the human drivers of greenhouse gas emissions and other environmental and ecological outcomes [3], [10], [11], [12], [13], [19], [21], [23]. The interpretation of estimated coefficients in elasticity models is straightforward. Specifically, the elasticity coefficient for each independent variable in such a model is the estimated percentage change in the dependent variable associated with a one percent increase in the independent variable, controlling for all other factors in the model.

**Table 2:** Elasticity coefficients for the regression of total carbon dioxide emissions in 85 nations, 1960–2005: two-way fixed effects model estimates.

	Model 1	Model 2
Total Population	1.55***	1.91**
	(13.20)	(9.79)
Total Population * Africa		−.58***
		(3.59)
Total Population * Asia		−.03
		(.17)
Total Population * Latin America		−.08
		(.46)
Gross Domestic Product Per Capita	.76***	.69***
	(16.35)	(15.73)
Gross Domestic Product Per Capita Squared	.02	.03
	(1.59)	(1.74)
Urban Population as Percent of Total Population	.71***	.85***
	(6.94)	(8.18)
Trade as Percent of Total Gross Domestic Product	.15***	.12*
	(3.22)	(2.51)
R-sq overall	.99	.99
N	850	850
estimated coefficients	99	102

Notes:

*** p<.001.

** p<.01.

* p<.05 (two-tailed); absolute values of z-ratios in parentheses; all models include unreported unit-specific and period-specific intercepts; all predictor variables and the outcome variable are in logarithmic form.

The findings for Model 1 indicate that a 1% increase in population size leads to a 1.55% increase in carbon emissions, net of the control variables and case-specific and time-specific fixed effects. This effect is also statistically significant at the .001 level. With the exception of GDP per capita squared, the estimated effects of the control variables are all generally consistent with established areas of research. The empirical evidence from prior research concerning a Kuznets distribution between emissions and development is mixed [25], [26]. However, for purposes of inclusivity and validity, we choose to include GDP per capita squared as a control variable in all estimated panel models. Turning to Model 2, the results indicate that the estimated effect of population size on carbon dioxide emissions differs for nations in Africa relative to nations in other regions. The estimated effect of population size for the Africa nations is .97, which we derive from adding the estimated elasticity coefficient for the statistically significant interaction between population size and Africa (−.58) to the estimated effect for the reference category (1.91). All other interactions between population size and region are nonsignificant, indicating no observed differences in the effect of population size on emissions in Asia and Latin America relative to the reference group (Europe, North America, Oceania). In other words, for nations in Africa, a 1% increase in population size leads to a .97% increase in carbon emissions, slightly less than a proportional relationship. For nations in all other regions, a 1% increase in population size leads to a 1.91% increase in carbon emissions, a more than proportional relationship. The r-square value for both models is .99, suggesting that both models explain 99 percent of variation in national-level carbon emissions. Such high r-square values are partly a function of the case-specific and period-specific intercepts that serve as fixed effects, which combined also allow for more rigorous hypothesis testing, and they reduce the chance of committing a type 1 error [24]. While these results highlight some notable regional differences in the emissions/population relationship, the estimated models do not assess if the effects of population size on emissions change in value through time. We now turn to the findings for the final series of analyses, which focus on such temporal dynamics for the entire sample as well as for the regional samples of nations.

**Figure 1:**
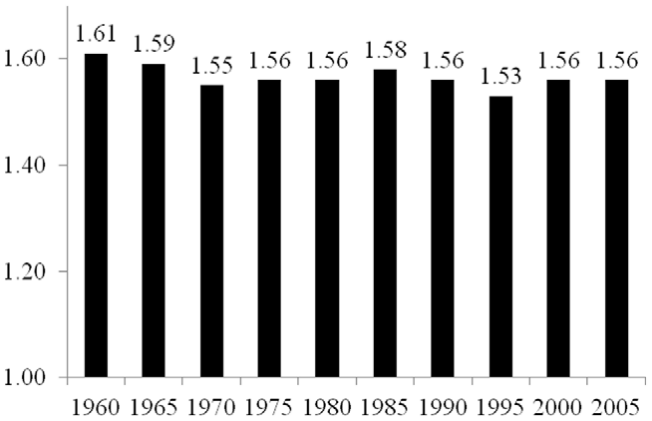
Estimated Effects of Population Size on Carbon Dioxide Emissions for 85 Countries, 1960–2005. Notes: effects (elasticity coefficients) are estimated using two-way fixed effects Prais-Winston regression with panel corrected standard errors and an ar(1) correction, with additional control variables. Derived from Model 1 in Table 3.

**Figure 2:**
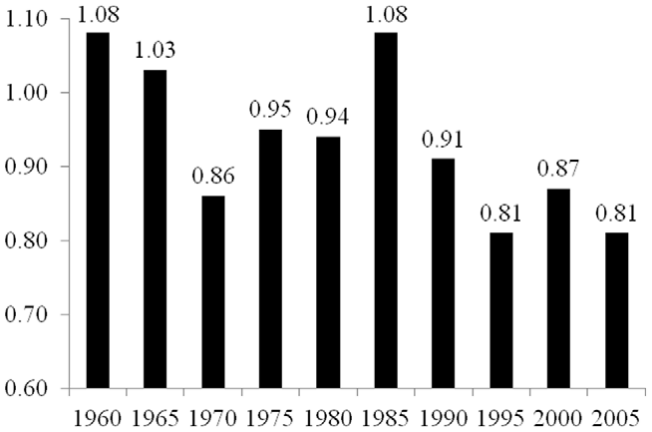
Estimated Effects of Population Size on Carbon Dioxide Emissions for 28 Countries in Africa, 1960–2005. Notes: effects (elasticity coefficients) are estimated using two-way fixed effects Prais-Winston regression with panel corrected standard errors and an ar(1) correction, with additional control variables. Derived from Model 2 in Table 3.

**Figure 3:**
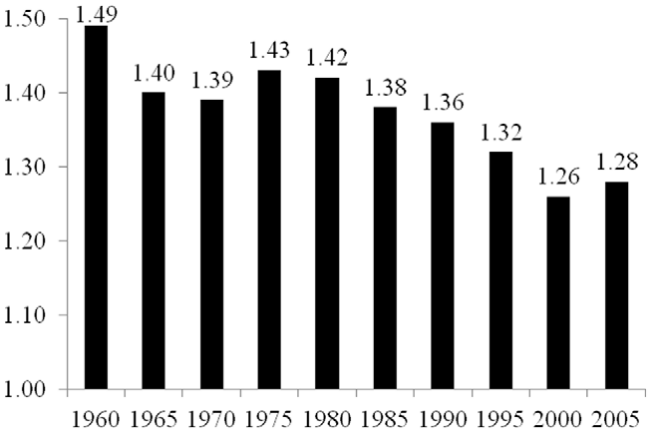
Estimated Effects of Population Size on Carbon Dioxide Emissions for 15 Countries in Asia, 1960–2005. Notes: effects (elasticity coefficients) are estimated using two-way fixed effects Prais-Winston regression with panel corrected standard errors and an ar(1) correction, with additional control variables. Derived from Model 3 in Table 3.

**Figure 4:**
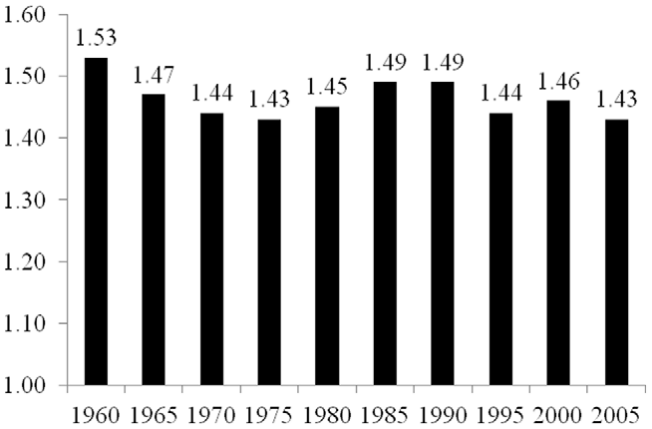
Estimated Effects of Population Size on Carbon Dioxide Emissions for 18 Countries in Latin America, 1960–2005. Notes: effects (elasticity coefficients) are estimated using two-way fixed effects Prais-Winston regression with panel corrected standard errors and an ar(1) correction, with additional control variables. Derived from Model 4 in Table 3.

**Figure 5:**
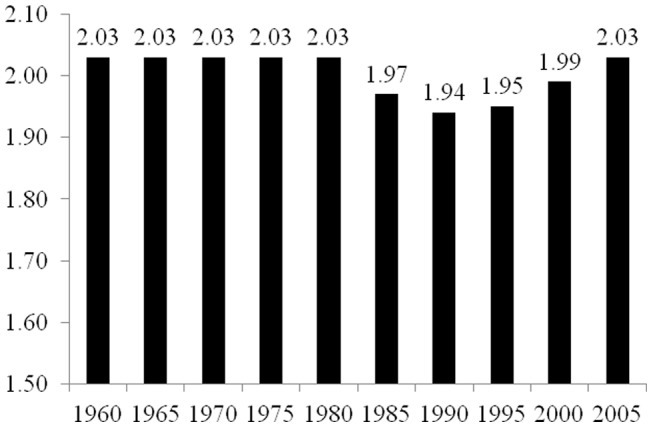
Estimated Effects of Population Size on Carbon Dioxide Emissions for 24 Countries in Europe, North America, and Oceania 1960–2005. Notes: effects (elasticity coefficients) are estimated using two-way fixed effects Prais-Winston regression with panel corrected standard errors and an ar(1) correction, with additional control variables. Derived from Model 5 in Table 3.

As described in the Materials and Methods section below, the elasticity coefficients reported in Figures 1, 2, 3, 4, and 5 are derived from two-way fixed effects panel regression models that include slope-dummy interactions between population size and dummy variables for each time point as well as additional control variables. Table 3 provides the full results for each of the five estimated models. The estimated effects of the control variables in all five models are relatively consistent with common findings for prior research on the human drivers of national-level carbon emissions [1], and like the analyses reported in Table 2, the very high r-square values for the five models are partly a function of the estimated two-way fixed effects.

**Table 3:** Elasticity coefficients for the regression of total carbon dioxide emissions in 85 nations, 1960–2005: two-way fixed effects model estimates.

	Model 1	Model 2	Model 3	Model 4	Model 5
Total Population	1.61***	1.08**	1.49***	1.53***	2.03***
	(12.86)	(2.79)	(4.62)	(8.30)	(9.11)
Total Population * 1965	−.02***	−.05**	−.09***	−.06***	−.01
	(4.37)	(2.67)	(10.89)	(7.92)	(.53)
Total Population * 1970	−.06***	−.22***	−.10***	−.09***	.01
	(8.91)	(7.77)	(10.91)	(13.45)	(.05)
Total Population * 1975	−.05***	−.13***	−.06***	−.10***	−.01
	(6.32)	(3.47)	(4.85)	(13.49)	(1.01)
Total Population * 1980	−.05***	−.14**	−.07***	−.08***	−.03
	(6.99)	(2.96)	(4.21)	(9.50)	(1.85)
Total Population * 1985	−.03***	−.04	−.11***	−.04***	−.06**
	(3.91)	(.76)	(5.27)	(6.03)	(3.17)
Total Population * 1990	−.05***	−.17**	−.13***	−.04***	−.09***
	(4.71)	(2.81)	(5.51)	(4.63)	(4.56)
Total Population * 1995	−.08***	−.27***	−.17***	−.09***	−.08***
	(5.83)	(3.89)	(5.58)	(9.05)	(3.69)
Total Population * 2000	−.05***	−.21***	−.23***	−.07***	−.04*
	(3.53)	(3.36)	(6.50)	(6.59)	(2.16)
Total Population * 2005	−.05***	−.27***	−.21***	−.10***	−.03
	(3.44)	(4.15)	(5.48)	(8.08)	(1.77)
Gross Domestic Product Per Capita	.78***	1.68***	.63***	.41***	.73***
	(16.07)	(6.51)	(6.89)	(4.96)	(6.05)
Gross Domestic Product Per Capita Squared	.02	.27***	−.01	−.06	.03
	(1.52)	(3.43)	(.74)	(1.25)	(1.01)
Urban Population as Percent of Total Population	.68***	.67***	.85***	1.40***	1.14***
	(6.60)	(3.35)	(7.23)	(6.94)	(5.94)
Trade as Percent of Total Gross Domestic Product	.16**	.21	.01	.13*	.20*
	(3.11)	(1.94)	(.01)	(2.11)	(2.12)
R-sq overall	.99	.96	.99	.99	.99
N	850	280	150	180	240
estimated coefficients	108	51	38	41	47

Notes: Model 1 is for all 85 countries; Model 2 is for the 28 countries in Africa; Model 3 is for the 15 countries in Asia; Model 4 is for the 18 countries in Latin America; Model 5 is for the 24 countries in Europe, North America, and Oceania;

*** p<.001.

** p<.01.

* p<.05 (two-tailed); absolute values of z-ratios in parentheses; all models include unreported unit-specific and period-specific intercepts; all predictor variables and the outcome variable are in logarithmic form.

Figure 1 provides the estimated elasticity coefficients of population size for each of the five-year time points for the overall sample of 85 countries based on Model 1 in Table 3. Overall, we find that the estimated effect of population size slightly decreases from 1960 to 2005. In 1960, a 1% increase in population size leads to a 1.61% increase in carbon emissions, and in 2005, a 1% increase in population size leads to a 1.56% increase in total emissions. However, the effect of population size on national-level carbon emissions is slightly smaller in 1970 (elasticity coefficient = 1.55) and 1995 (elasticity coefficient = 1.53) than in 2005. While these findings in general suggest a decrease in the effect of population size on carbon emissions, the decrease in the size of the effect for the entire sample of nations is relatively small. Overall, population size continues to be an important and primary contributor of national-level total carbon dioxide emissions.

Figure 2 reports the estimated elasticity coefficients of population size for each time point for the sample of 28 countries in Africa, based on Model 2 in Table 3. In general, we find a moderate reduction in the estimated effect of population size on national-level carbon dioxide emissions. For this sample, in 1960 a 1% increase in population size leads to a 1.08% increase in emissions, while in 2005, a 1% increase in population size leads to a .81% increase in carbon dioxide emissions. This overall reduction is much more pronounced than the observed decrease in the analyses of all countries in Figure 1. However, during the 45-year period of investigation, the estimated effect of population size appeared to decrease in size initially, followed by an upward trend during the mid-1970s to mid-1980s, with the estimated effect of population size on emissions in 1985 returning to the same value as in 1960 (i.e., elasticity coefficient = 1.08). From 1985 through 2005 the estimated effect of population size decreased to an elasticity coefficient of .81.

The findings presented in Figure 3, which are derived from Model 3 in Table 3, indicate that for the sample of 15 countries in Asia, and similar to the analysis of the countries in Africa, the estimated effect of population size on national-level carbon dioxide emissions decreased moderately in value from 1960 to 2005. In 1960, a 1% increase in population size leads to a 1.49% increase in emissions, while in 2005, a 1% increase in population size leads to a 1.28% increase in national-level total carbon dioxide emissions. With few exceptions (i.e., 1970 to 1975, 2000 to 2005), the effect of population size on total emissions decreased successively at each 5-year time point.

Figure 4 reports the estimated effects of population on national-level total carbon dioxide emissions for each time point for the sample of 18 countries in Latin America, based on Model 4 in Table 3. While the analysis suggests a decline from 1960 to 2005 in the size of the estimated elasticity coefficient for population size, the magnitude of the decrease is relatively modest compared to the observed declines for the samples of countries in Africa and in Asia. For the Latin America sample, in 1960, a 1% increase in population size leads to a 1.53% increase in national-level emissions, while in 2005, a 1% increase in population size leads to a 1.43% increase in national-level carbon dioxide emissions. Relatively similar to the analysis of the countries in Africa, the estimated elasticity coefficient for population size decreased successively through the 1970s, followed by increases through the late 1980s and early 1990s, then generally declined through 2005. However, these downward and upward patterns are much more modest than those observed for the Africa sample of nations.

The final analysis for the study is reported in Figure 5 and derived from Model 5 in Table 3, which involves the estimated effects of population size on national-level total emissions for each time point for the sample of 24 countries in Europe, North America, and Oceania. Unlike the analyses presented in Figures 1, 2, 3, and 4, the results here indicate that the estimated elasticity coefficient for population size is the same value in 2005 as in 1960. For these beginning and ending periods of the study, in this sample of nations a 1% increase in population size leads to a 2.03% increase in total carbon dioxide emissions. Further, the estimated elasticity coefficient has a value of 2.03 from 1960 through 1980, followed by lesser values of 1.97 in 1985 and 1.94 in 1990, and then increasing values of 1.95 in 1995, 1.99 in 2000, and 2.03 in 2005. More generally, it appears that for all other regions, the relationship between national-level carbon dioxide emissions and total population size decoupled to some extent from 1960 to 2005, while for this region, which consists of mainly high-income nations, the national-level emissions/population relationship remained relatively time-invariant in magnitude for the overall 45-year period of investigation.

## Discussion

Like past research on national-level anthropogenic emissions [1], [2], [3], [10], [12], [13], [14], [15], [19], this study shows that overall population size is a primary driver of total carbon emissions in cross-national contexts. However, unlike past research, the results of this study indicate that the national-level relationships between total anthropogenic carbon dioxide emissions and population size include regional differences and are temporally dynamic. All regions experienced overall increases in carbon dioxide emissions and population size during the 45-year period of investigation, but at notably different rates. For carbon dioxide emissions, the sample of 15 countries in Asia experienced a 698% increase, the 18 countries in Latin America experienced a 443% increase, the 28 countries in Africa experienced a 408% increase, and the 24 countries in Europe, North America, and Oceania experienced a 117% increase. For population size, the sample of countries in Africa experienced a 215% increase, the sample of Latin American countries experienced a 157% increase, the sample of countries in Asia experienced a 124% increase, and the sample of countries in Europe, North America, and Oceania experienced a 53% increase. The initial cross-national two-way fixed effects panel regression analysis of national-level carbon dioxide emissions that included additional explanatory variables suggested that the estimated elasticity coefficient for population size is much smaller for nations in Africa than for nations in other regions of the world. The final series of cross-national panel regression analyses that also included additional control variables and two-way fixed effects suggested that from 1960 to 2005, the estimated elasticity coefficient for population size decreased by 25% for the sample of Africa countries, decreased by 14% for the sample of Asia countries, decreased by 6.5% for the sample of Latin America countries, but remained the same for the sample of relatively high-income countries in Europe, North America, and Oceania.

Thus, while significant attention should continue to be given to the rapid rates of population growth and total carbon dioxide emissions in the developing nations in regions other than in Europe, North America, and Oceania, it is also necessary to consider these relationships in the developed nations, even though their growth rates for population size and carbon emissions might be lower than in the other regions. This research indicates that population still matters and continues to be a primary driver of national-level total anthropogenic carbon dioxide emissions. Beyond this, the study highlights that the actual impacts of population size and growth on national-level carbon dioxide emissions are far from monolithic, both across regions and through time. There are important regional and temporal variations, and it is critical to employ appropriate and rigorous model estimation techniques that include other relevant independent variables and that also account for heterogeneity bias through the use of case-specific and time-specific fixed effects. Our study also shows how one can employ rigorous model estimation techniques that provide robust and easily interpretable results.

The recent Royal Society report [9] as well as related documents of other groups, such as the U.S. National Research Council [7], [8], propose that population and the environment cannot be seen as separate issues, especially given that the global population will continue to increase for at least the next few decades. They also suggest that demographic and population concerns in general must be included within any discussions of economic and environmental sustainability, including assessments and discussions of human drivers of greenhouse gas emissions and climate change. Our research indicates that future policies and programs aimed at carbon dioxide emissions mitigation would do well to recognize regional differences in national-level total carbon emissions and population size relationships, and especially their temporal dynamics. These relationships and temporal patterns influence national, regional, and global environmental conditions and ultimately the earth's carrying capacity [27].

## Materials and Methods

For this study we used national level data obtained from the World Bank's World Development Indicators [28]. The 85 countries included in the analyses are listed in Table 1. The total carbon dioxide emissions data, measured in metric tons, represent the mass of carbon dioxide produced during the combustion of solid, liquid, and gaseous fuels, as well as from gas flaring and the manufacture of cement. They do not include emissions from land use change or emissions from bunker fuels used in international transportation. The total population size data are based on the de facto definition of population, which counts all residents regardless of legal status or citizenship. Refugees not permanently settled in the country of asylum are generally considered to be part of the population of their country of origin. In the panel regression analyses we include gross domestic product (GDP) per capita and its quadratic (centered to reduce extreme collinearity), urban population as percent of total population, and trade as percent of total GDP as statistical controls. Like population size, these variables are consistently found in prior research to influence the carbon dioxide emissions levels of nations [1], [2], [3], [10], [11], [12], [13], [14], [15], [21], [23]. The GDP per capita data are measured in 2000 constant U.S. dollars. As noted in the results section, for the panel regression analyses the dependent variable and all independent variables are in logarithmic form and thus the regression models estimate elasticity coefficients [22], [29]. The coefficients of an elasticity model are relatively easy to interpret. Specifically, the coefficient for each independent variable in such a model is the estimated percentage change in the dependent variable associated with a 1% increase in the independent variable, controlling for all other factors in the model.

For the panel regression analyses we use a time-series cross-sectional Prais-Winsten (PW) regression model with panel-corrected standard errors (PCSE), allowing for disturbances that are heteroskedastic and contemporaneously correlated across panels. We employ PCSE because the feasible generalized least-squares estimator that is often used to analyze panel data produces standard errors that can lead to extreme overconfidence with panel datasets that do not have many more time periods than panels [30]. We correct for AR(1) disturbances (i.e., first-order autocorrelation) within panels, and since we have no theoretical basis for assuming the process is panel specific, we treat the AR(1) process as common to all panels [30]. We control for both period-specific and unit-specific disturbances. The general model is as follows:
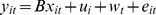
Subscript *i* represents each unit of analysis (i.e., country), subscript *t* represents the time period, and *y_it_* is the dependent variable for each country at each time period. *Bx_it_* represents the vector of coefficients for predictor variables that vary over time, *u_i_* is the unit-specific (i.e., country-specific) disturbance term, *w_t_* is the period-specific disturbance term that is constant across all countries, and *e_it_* is the disturbance term unique to each country at each point in time. We calculate and employ dummy variables to control for *u_i_* and *w_t_*. The former controls for potential unobserved heterogeneity that is temporally invariant within countries (unit-specific intercepts), while the latter controls for potential unobserved heterogeneity that is cross-sectionally invariant within periods (period-specific intercepts). The unit-specific intercepts approach is analogous to the dummy variable fixed effects model often referred to as one-way fixed effects [24]. Similarly, the inclusion of the period-specific intercepts is equivalent to modeling temporal fixed effects, and including both period-specific and unit-specific intercepts is analogous to estimating a two-way fixed effects model [31]. Including period-specific intercepts also lessens the likelihood of biased model estimates resulting from outcomes and predictors with relatively similar time trends [22]. Overall, our modeling approach is robust against potentially omitted control variables and more closely approximates experimental conditions than other panel model approaches [32]. The panel analyses are conducted with the “xtpcse” suite of commands in Stata (version 11) software.

For the panel regression analyses reported in Table 2, we estimated the following two models for the full sample of 85 countries:

**Model 1 of** ***Total Carbon Dioxide Emissions_it_*** **= **

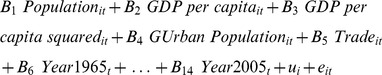



**Model 2 of** ***Total Carbon Dioxide Emissions_it_*** **= **

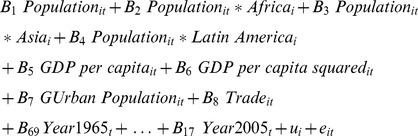
Thus, Model 1 includes total population, GDP per capita, GDP per capita squared, urban population, trade as percent of GDP, and the period-specific intercepts (i.e., 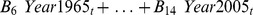) as well as the country-specific intercepts and the disturbance term unique to each country at each point in time. Model 2 also includes slope dummy interactions between population size and dummy variables for region (labeled Africa, Asia, Latin America), where the combined region of Europe, North America, and Oceania serve as the reference category. We remind readers that the time-invariant dummy variables for region are perfectly correlated with the country-specific fixed effects and thus excluded as main effects in the estimated model [24].

The results reported in Table 3 as well as Figure 1 for the entire sample of nations and in Figures 2, 3, 4, and 5 for the region-specific national samples are derived from the following estimated model:

***Total Carbon Dioxide Emissions_it_*** **= **

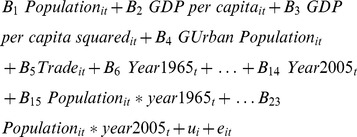
This model builds on Model 1 in Table 2 above, but also includes slope dummy interactions between population size and the dummy variables for each year (i.e., 1965, 1970, …, 2000, 2005) where the year 1960 is the reference category. The interactions between population size and the year dummy variables allow us to assess the extent to which the estimated effect of population on total carbon dioxide emissions increases or decreases through time. For these estimated models that include the interactions between population size and time, the elasticity coefficient for total population is the unit change in the dependent variable in 1960 for each unit increase in total population for the same year. Since all continuous variables are in logarithmic form in the estimated models, units here correspond to percentages. The overall effect of total population for the other time points (i.e., 1965, 1970, 1975, 1980, 1985, 1990, 1995, 2000, 2005) equals the sum of the elasticity coefficient for total population (i.e., its effect in 1960) and the appropriate interaction term if the latter is statistically significant. A nonsignificant coefficient for the latter suggests that there is no difference in the effect of population size in 1960 and the later time point. This technique is well established in cross-national longitudinal research on the human drivers of greenhouse gas emissions as well as research on other topics [10], [19], [20], [23], [24], [33], [34], [35], [36], [37], [38].
